# Expression profiles of VEGF-A, VEGF-D and VEGFR1 are higher in distant metastases than in matched primary high grade epithelial ovarian cancer

**DOI:** 10.1186/s12885-019-5757-3

**Published:** 2019-06-14

**Authors:** Minna Sopo, Maarit Anttila, Kirsi Hämäläinen, Annukka Kivelä, Seppo Ylä-Herttuala, Veli-Matti Kosma, Leea Keski-Nisula, Hanna Sallinen

**Affiliations:** 1 Department of Gynecology, Kuopio University Hospital, Kuopio, Finland; 2 Department of Pathology and Forensic Medicine, Kuopio University Hospital, Kuopio, Finland; 3 Department of Biotechnology and Molecular Medicine, A.I.Virtanen Institute for Molecular Sciences, University of Eastern Finland, Kuopio, Finland; 4 Institute of Clinical Medicine, School of Medicine, Gynaecology, University of Eastern Finland, Kuopio, Finland; 5 Pathology and Forensic Medicine, University of Eastern Finland, Kuopio, Finland; 6 Cancer Center of Eastern Finland, University of Eastern Finland, Kuopio, Finland

**Keywords:** Ovarian cancer, Immunohistochemistry, Angiogenesis, VEGF, VEGF receptors

## Abstract

**Background:**

In many malignancies including ovarian cancer, different angiogenic factors have been related to poor prognosis. However, data on their relations to each other or importance as a prognostic factor in ovarian cancer is missing. Therefore, we investigated the expressions of VEGF-A, VEGF-C, and VEGF-D, and the receptors VEGFR1, VEGFR2, and VEGFR3 in patients with malignant epithelial ovarian neoplasms. We further compared expression levels between primary tumors and related distant omental metastases.

**Methods:**

This study included 86 patients with malignant ovarian epithelial tumors and 16 related distant metastases. Angiogenic factor expression was evaluated using immunohistochemistry (*n* = 102) and qRT-PCR (*n* = 29).

**Results:**

Compared to primary high grade serous ovarian tumors, the related omental metastases showed higher expressions of VEGF-A (*p* = 0.022), VEGF-D (*p* = 0.010), and VEGFR1 (*p* = 0.046). In univariate survival analysis, low epithelial expression of VEGF-A in primary tumors was associated with poor prognosis (*p* = 0.024), and short progression-free survival was associated with high VEGF-C (*p* = 0.034) and low VEGFR3 (*p* = 0.002). The relative expressions of VEGF-D, VEGFR1, VEGFR2, and VEGFR3 mRNA determined by qRT-PCR analyses were significantly correlated with the immunohistochemically detected levels of these proteins in primary high grade serous ovarian cancer and metastases (*p* = 0.004, *p* = 0.009, *p* = 0.015, and *p* = 0.018, respectively).

**Conclusions:**

The expressions of VEGF receptors and their ligands significantly differed between malignant ovarian tumors and paired distant metastases. VEGF-A, VEGF-D, and VEGFR1 protein expressions seem to be higher in distant metastases than in the primary high grade serous ovarian cancer lesions.

## Background

Among gynecological cancers, ovarian cancer has the worst prognosis and leads to over 150,000 deaths worldwide each year [1]. At the time of diagnosis, 70% of ovarian cancers are disseminated. Standard treatment strategies include cytoreductive surgery and platinum based chemotherapy. Even with optimal therapy, 30–50% of patients die within 5 years after diagnosis [2]. There are presently no curative treatment options for recurrent disease. Prognostic markers for ovarian cancer include tumor stage, residual tumor after primary surgery, the primary response to platinum-based chemotherapy, tumor grade, histological type, and patient age [3].

Improved knowledge of cancer growth and dissemination mechanisms at the molecular level has enabled targeted treatments. In ovarian cancer, targeting endothelial cells of the tumor blood vessels is already standard therapy in both recurrent and primary disease [4, 5]. Additionally, phase I–III studies are presently underway to investigate new antiangiogenic treatment options. Thus, there is an urgent need for biomarkers predicting patient prognosis and responses to specific therapies, to accelerate the development of more personalized treatments [6–8].

VEGF (vascular endothelial growth factor) signaling pathways play major roles in tumor angiogenesis and lymphangiogenesis [9, 10]. VEGF-A, -B, -C, and -D and PlGF signal through three tyrosine kinase receptors: VEGFR1, 2, and 3. VEGF-A binds to VEGFR1 and VEGFR2 and is the main stimulator of tumor growth and dissemination. VEGF-A/VEGFR2 signaling is considered the most important pathway for tumor angiogenesis, inducing endothelial permeability and stimulating the accumulation of malignant ascites in ovarian cancer patients [11]. VEGF-C and -D signal mainly through VEGFR3, stimulating lymphangiogenesis and contributing to the generation of angiogenic sprouts [10, 12–15].

It is essential to evaluate the behavior of angiogenic factors, and to develop new biomarkers for accurate diagnosis and prognosis. We previously reported that alterations of angiogenesis-related circulating proteins—such as elevated angiopoietin-2, high Ang-2/sVEGFR2 ratio, and low level of VEGFR2—predict poor overall survival (OS) and progression-free survival (PFS) in ovarian cancer patients [10]. Earlier studies have shown that VEGF, VEGFR2, and VEGFR3 expressions are related to poor prognosis in endometrial, colorectal, and lung adenocarcinoma [16–18]. In ovarian carcinoma, VEGFR1 expression is reportedly associated with shorter PFS [19]. To date, no studies have evaluated all VEGFs and their receptors at the tumor tissue level in both primary tumors and related metastases.

In our present study, we performed immunohistochemical evaluation of VEGF-A, −C, and -D and VEGFR1, R2, and R3 in 86 ovarian carcinoma primary tumors and 16 related metastatic tumors. We measured the expressions of these angiogenic factors using qRT-PCR of 29 tumors. Our aims were to determine whether the expression levels differed between primary tumors and the related metastases, and whether expression of these factors predicted the clinical course, prognosis, and survival of the epithelial ovarian carcinoma patients. To our knowledge, this is the first study to evaluate all VEGFs and their receptors, using both immunohistochemistry and qRT-PCR, in primary ovarian tumors and related metastatic tumors from the same patients, and to correlate the expression data with clinical outcomes.

## Methods

### Patients

This study included a total of 86 women who were diagnosed with malignant ovarian epithelial tumors at Kuopio University Hospital between 1999 and 2007. Tumor tissue samples and patient information were prospectively collected at the time of primary diagnosis, and retrospectively analyzed using immunohistochemistry and qRT-PCR. The follow-up time ended in March 2017. In a subgroup of 16 patients with high grade serous ovarian carcinoma, we also analyzed related metastases. The metastatic samples were omental metastases at the time of primary diagnosis. The patient ages ranged from 29 to 88 years (median, 58 years). Histological type and grade were evaluated according to the World Health Organization (WHO) Classification of Tumors [20]. Patients with nonepithelial neoplasms, patients treated prior to operation, unoperated patients (totally 65 patients) were excluded from this study. Epithelial ovarian carcinomas were operatively staged according to the International Federation of Gynecology and Obstetrics (FIGO) criteria. All included patients were treated with platinum-based chemotherapy. Table 1 summarizes the patient characteristics. This study was approved by the Ethical Committee of the Kuopio University Hospital [10].

**Table 1:** Clinicopathological data of the ovarian cancer patients

Variable	Ovarian carcinoma (%)
Total	86 (100)
Median age at diagnosis, years	58 (26–88)
Histologic subtype	
Serous	51 (59)
High grade	45
Low grade	6
Mucinous	11 (13)
Endometrioid	15 (17)
High grade	13
Low grade	2
Clear cell	5 (6)
Other	4 (5)
Ca12–5 median, kU/l	363 (5–10,100)
FIGO Stage	
I	12 (13)
II	10 (12)
III	46 (53)
IV	18 (22)
Histological grade^a^	
1	9 (12)
2	25 (33)
3	41 (55)
Ascites	60 (70)
No ascites	16 (19)
No data on ascites	10 (11)
Residual tumor at primary surgery	
None	40 (47)
≤ 1 cm	8 (9)
> 1 cm	38 (44)
Chemotherapy response	
Complete response	57 (66)
Partial response	4 (5)
Stable disease	2 (2)
Progressive disease	5 (6)
No chemotherapy	5 (6)
No data	13 (15)
Tumor recurrence	42 (49)
No recurrence	25 (29)
No data on recurrence	19 (22)
Patient status	
Dead from ovarian cancer	46 (53)
Alive	35 (41)
Median follow-up, months	65 (0–198)

Values are presented as *n* (%) or as indicated units (range)

^a^ Mucinous tumors are not graded

### Immunohistochemistry

Tissue samples were embedded in paraffin and cut into 5-μm-thick sections. Next, the sections were processed for staining with hematoxylin-eosin, VEGF-A (1:250; Santa Cruz), VEGF-C (1:20; Novus), VEGF-D (1:20; Santa Cruz), VEGFR1 (1:15; Santa Cruz), VEGFR2 (1:750; Cell Signaling), and VEGFR3 (11:000, Millipore/Chemicon) [21–25].

During evaluation of immunostained sections, the investigators were blinded to the patients’ clinical status. Immunostaining of VEGF-A, VEGF-C, VEGF-D, VEGFR1, VEGFR2, and VEGFR3 was microscopically evaluated (Leitz Wetzlar 512,761/20; Germany). After screening the whole section, ten randomly selected microscopic fields were examined at × 200 magnification (× 20 objective lens and × 10 ocular lens; 0.74 mm^2^ per field) to determine the mean percentage of specific immunostained epithelial tumor cells. The percentage of positively stained cells (PP) was assigned as a numerical score: 0, negative; 1, < 10%; 2, 11–50%; 3, 51–80%; and 4, > 80% positive cells. The intensity (SI) of the immunostained areas was defined as follows: 0, negative; 1, weak; 2, moderate; 3, strong. An immunoreactive score (IRS) ranging from 0 to 12 was calculated using the following formula: IRS = PP × SI [26].

The percentage of strong epithelial staining was separately determined for the VEGFR1, 2, and 3 sections. Stromal immunostaining was evaluated by ranking the staining intensity: 0, negative; 1, weak; 2, strong. Endothelial expression was deemed positive or negative, and we also evaluated the cellular distribution of the staining (cytoplasm, nucleus, and cell membrane) in epithelial ovarian tumors. Stromal expression was positive in all samples, and thus the staining intensity was classified as weak or strong.

### Quantitative real-time polymerase chain reaction (qRT-PCR) analysis

We performed qRT-PCR analysis of samples from 13 primaries and 16 metastatic tumors.

RNA was isolated using TRI-reagent (Sigma Aldrich, St. Louis, Missouri, USA), and treated with DNase (Promega, Fitchburg, Wisconsin, USA). From 5 μg total RNA, cDNA was synthesized using random hexamer primers (Promega) and RevertAid™ reverse transcriptase (Fermentas, Waltham, Massachusetts, USA). We measured the relative expressions of the mRNAs encoding VEGF-A, VEGF-C, VEGF-D, VEGFR1, VEGFR2, and VEGFR3 by qRT-PCR using specific Assays-on-Demand target mixes (Applied Biosystems, Foster City, California, USA), following the manufacturer’s protocol (StepOnePlus, Applied Biosystems). The expression levels were normalized to peptidylprolyl isomerase A [25, 27].

### Statistical analysis

Statistical analyses were performed using SPSS for Windows (version 24, 1989–2016, SPSS Inc., Chicago, USA). We performed the Kruskal-Wallis test followed by the Mann-Whitney test with multiple comparisons when appropriate. For analyses of clinicopathological associations and survival analyses, PP values, IRS values, and PCR levels were each dichotomized into low and high groups using the median values as a cut-off [10]. We used the chi-squared test to analyze frequency tables, and the Wilcoxon signed-rank test to compare histology and qRT-PCR results between primary ovarian tumors and the related metastases. Univariate survival analyses were based on the Kaplan-Meier method, and survival curves were compared using the log-rank test. Variables that were significant in univariate analyses were entered in a stepwise manner into the Cox regression multivariate analysis. OS was defined as the time interval between the date of surgery and the date of death or the end of follow-up. PFS was defined as the time interval between the date of surgery and the date of identified recurrence or death. Correlations between histological parameters and qRT-PCR levels were analyzed using the Spearman’s correlation test. A *p* value of < 0.05 was considered significant [10].

## Results

### Immunohistochemical analyses of VEGF-A, VEGF-C, VEGF-D, VEGFR1, VEGFR2, and VEGFR3 in primary high grade serous and endometrioid ovarian tumors

**Fig. 1:**
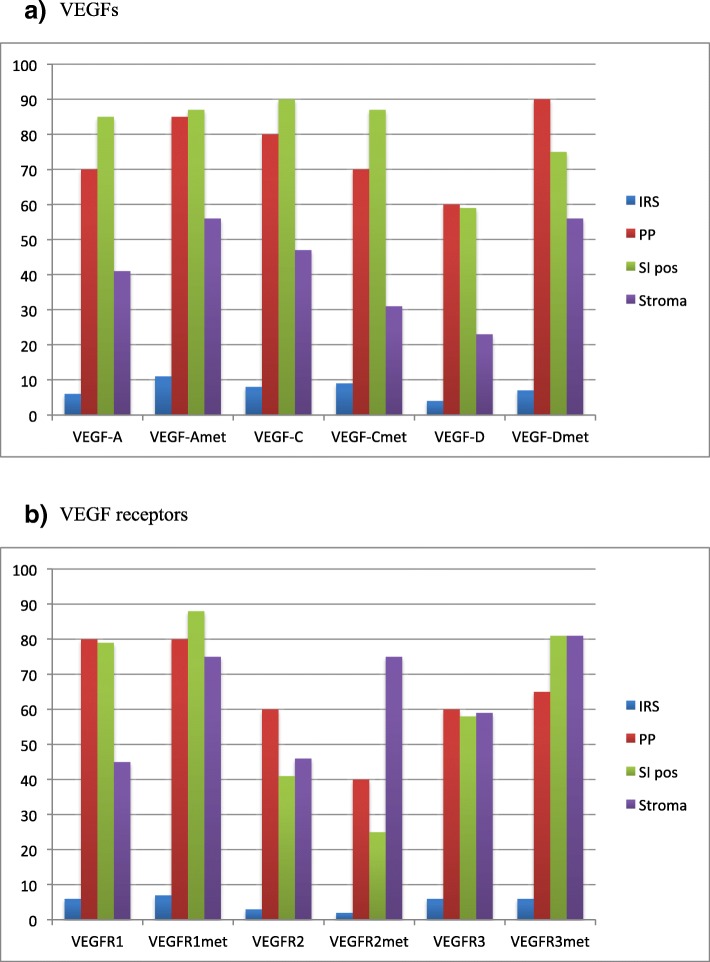
Diagram of the immunohistochemical parameters of primary tumors and metastases. **a**) VEGFs and **b**) VEGF-receptors. *IRS* immunoreactive score, *PP* percentage of positively stained cells, *SI* staining intensity

VEGF-A, VEGF-C, and VEGF-D were mainly detected in the cytoplasm of tumor epithelial cells. Of these VEGFs, VEGF-C showed the strongest expression in epithelial primary high grade serous and endometrioid ovarian cancer cells. VEGF-C staining intensity among epithelial cancer cells was moderate to strong in 89% of primary tumors (IRS, 8), while moderate-to-strong VEGF-A staining intensity was found in 86% of primary tumors (IRS, 6), and VEGF-D staining was at least moderate in 56% of primary tumors (IRS, 4). Of all evaluated factors, epithelial immunostaining of VEGF-D was the weakest. In primary high grade serous and endometrioid tumors, strong stromal staining was observed in 40% of VEGF-C-stained samples, 41% of VEGF-A-stained samples, and 21% of VEGF-D-stained samples. Stromal staining was most prominent in the stromal fibroblasts and endothelial staining was positive in all samples (Fig. 1).

**Fig. 2:**
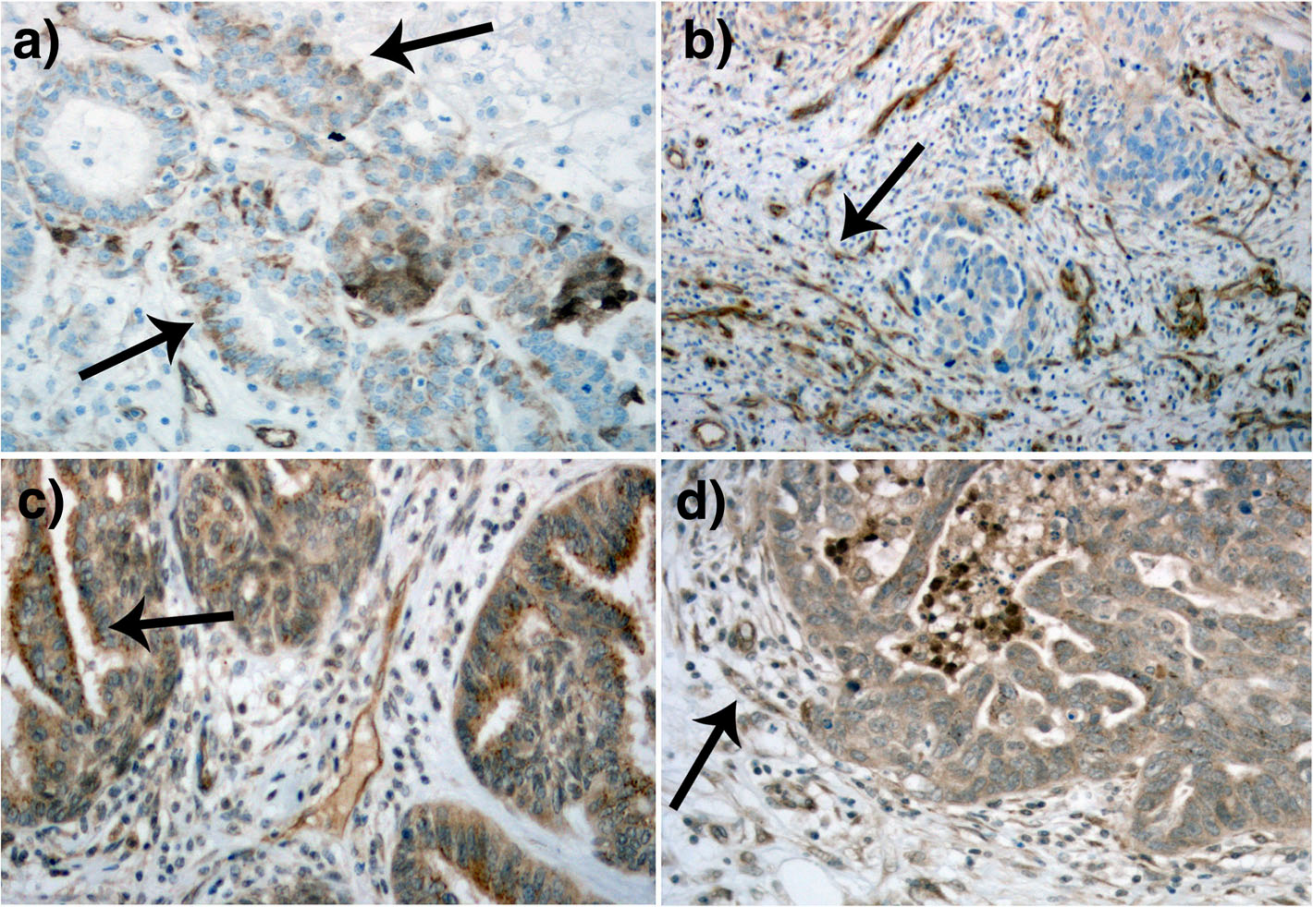
Images show VEGFR2 staining in a primary tumor samples (**a**) and metastasis sample (**b**), and VEGFR3 staining in a primary tumor samples (**c**) and metastasis sample (**d**). VEGF-receptors were mainly expressed in the epithelial cell membrane (**a** and **c**, black arrows), in stromal tissue and in vascular endothelial cells (**b** and **d**, black arrows)

VEGF receptors (R1, R2, R3) were mainly expressed in the epithelial cell membranes of primary tumors, in stromal tissue, and in vascular endothelial cells (Fig. 2). For VEGFR1, VEGFR2, and VEGFR3, respectively, the median IRS values were 6, 3, and 4; and the median PP values for epithelial cells were 75, 60, and 60%. Epithelial expression of VEGFR1 was strongest, while VEGFR2 was weakest. We detected strong stromal staining in 37% of VEGFR1-stained samples, 44% of VEGFR2-stained samples, and 65% of VEGFR3-stained samples in high grade serous and endometrioid cancer. Vascular endothelial staining was notably strong for all receptors in most samples (Fig. 2). Immunohistochemical analysis of all histologies and grades is presented in Table 2 and Fig. 1. There was no significant difference when high grade serous and endometrioid tumors were analyzed separately compared to the whole study population.

**Table 2:** Expressions of VEGFs and their receptors in primary ovarian carcinomas and related metastases

Variable	Primary tumor (*n* = 86)	Metastases (*n* = 16)	*p*	Variable	Primary tumor (*n* = 85)	Metastases (*n* = 16)	*p*
VEGF-A				VEGFR1			
IRS	6	10.5	ns	IRS	6	7	ns
PP	70	85	0.022	PP	80	80	ns
SI			ns	SI			0.046
0 or weak	13 (15%)	2 (13%)		Weak	18 (21%)	2 (12%)	
Moderate	45 (52%)	5 (31%)		Moderate	39 (46%)	8 (50%)	
Strong	28 (33%)	9 (56%)		Strong	28 (33%)	6 (38%)	
Stroma SI				Stroma SI			
Weak	51 (59%)	7 (44%)		Weak	47 (55%)	4 (25%)	
Strong	35 (41%)	9 (56%)		Strong	38 (45%)	12 (75%)	
VEGF-D				VEGFR2			
IRS	4	7	0.013	IRS	3	2	ns
PP	60	90	0.010	PP	60	40	ns
SI			ns	SI			ns
0 or weak	35 (41%)	4 (25%)		0 or weak	50 (59%)	12 (75%)	
Moderate	37 (43%)	7 (44%)		Moderate	29 (34%)	4 (25%)	
Strong	14 (16%)	5 (31%)		Strong	6 (7%)	0	
Stroma SI				Stroma SI			
Weak	66 (77%)	7 (44%)		Weak	46 (54%)	4 (25%)	
Strong	20 (23%)	9 (56%)		Strong	39 (46%)	12 (75%)	
VEGF-C				VEGFR3			
IRS	8	9	ns	IRS	6	6	ns
PP	80	70	0.010	PP	60	65	ns
SI			ns	SI			ns
Weak	8 (9%)	2 (13%)		Weak	36 (42%)	3 (19%)	
Moderate	46 (54%)	4 (25%)		Moderate	40 (47%)	9 (56%)	
Strong	31 (36%)	10 (62%)		Strong	9 (11%)	4 (25%)	
Stroma SI				Stroma SI			
Weak	45 (53%)	11 (69%)		Weak	35 (41%)	3 (19%)	
Strong	40 (47%)	5 (31%)		Strong	50 (59%)	13 (81%)	

IRS and PP are presented as median values, SI is presented as n (%)

*IRS* immunoreactive score, *PP* percentage of positively stained epithelial cells, *SI* staining intensity

**Fig. 3:**
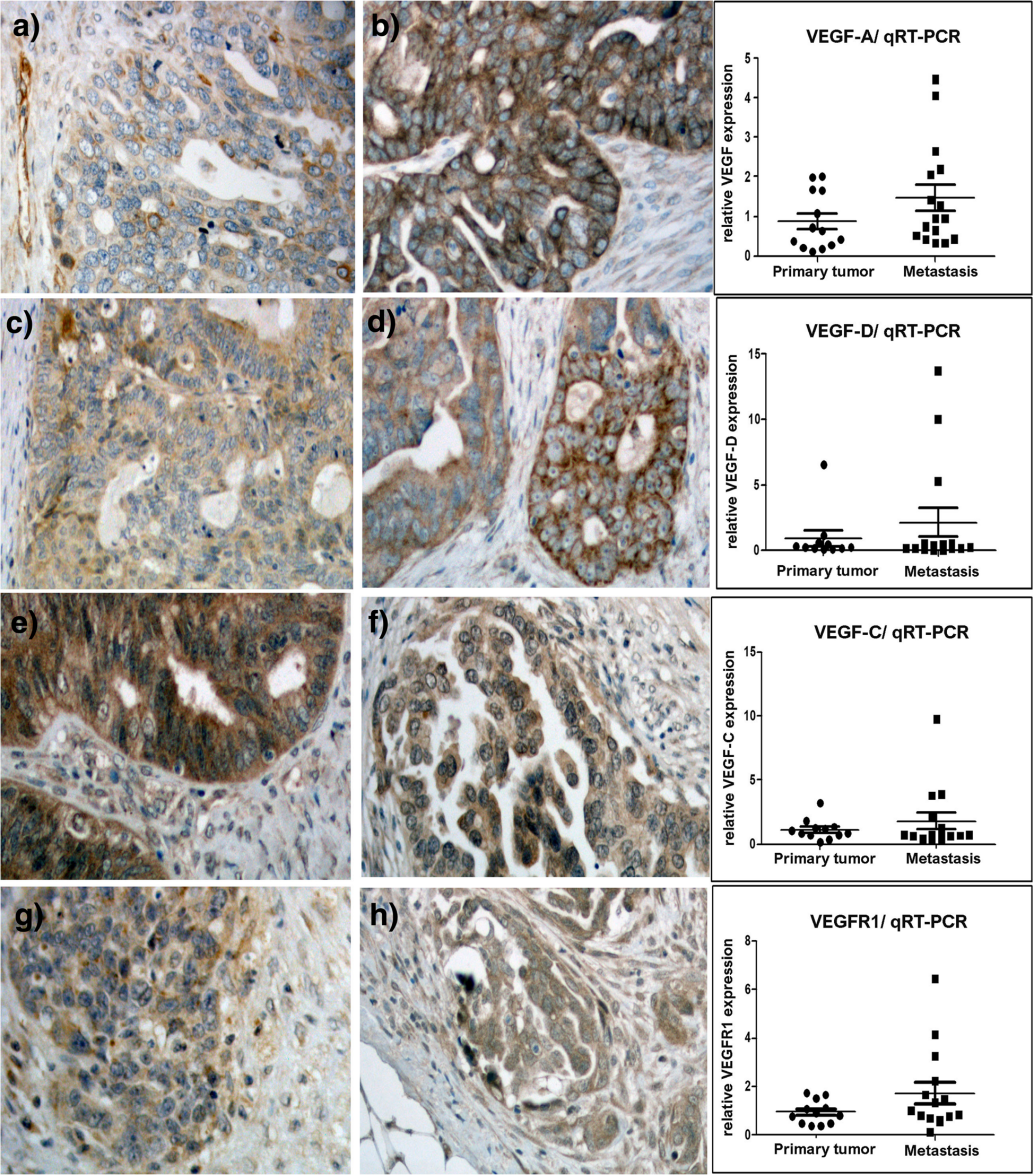
Compared to primary tumors, the related omental metastases showed stronger expressions of VEGF-A (**a** and **b**), VEGF-D (**c** and **d**), and VEGFR1 (**g** and **h**). Primary tumors are shown on the left, and metastases in the middle column. The right column presents the mRNA levels of the analogous angiogenic factors, as determined by qRT-PCR. **e** and **f** VEGF-C expression was higher in primary tumors than in metastases

In primary ovarian tumors, VEGFR3 showed moderate but significant correlations with its ligands lymphangiogenic VEGF-C (r = 0.4, *p* < 0.0005) and VEGF-D (r = 0.4, *p* = 0.001). Similarly, VEGFR2 was significantly correlated with VEGF-D (r = 0.4, *p* < 0.0005). In primary tumors, VEGF-A was weakly correlated with VEGFR2 (r = 0.2, *p* = 0.03), but was not significantly correlated with VEGFR1. We observed only weak correlations among VEGFs, while VEGF receptors were moderately correlated with each other in primary tumors. In high grade tumors the correlations did not differ significantly from the presented ones. (data not shown).

### Immunohistochemical expression of VEGF-A, VEGF-C, VEGF-D, VEGFR1, VEGFR2, and VEGFR3 in metastatic lesions of high grade serous ovarian cancer

In metastatic epithelium and stroma, the tumor tissue distribution of VEGF-A, VEGF-C, and VEGF-D expression paralleled the expression pattern in the primary cancer (Fig. 1). Of all VEGFs, VEGF-A expression was the strongest in tumor epithelium. VEGF-D was expressed in a wide area, but its staining intensity was weak. Among the growth factors, VEGF-A and VEGF-D dominated the stromal expression in metastases. (Table 2, Fig. 1). As in the primary tumors, metastases showed strong growth factor staining in vascular endothelium.

In metastases, we observed strong receptor staining in stromal tissue and vascular endothelium (Fig. 2). VEGFR1 exhibited the strongest epithelial cell expression, and VEGFR2 the weakest expression in ovarian cancer metastases (Table 2, Fig. 1). VEGF-A was significantly correlated with VEGFR1 in metastases (r = 0.502, *p* = 0.048).

### Angiogenic factor expression in primary high grade tumors compared to the related metastases

VEGF-A and VEGF-D expressions in epithelial tumor cells were significantly stronger in omental metastases than in the related primary tumors. Compared to primary tumors, metastases showed a significantly higher median PP of VEGF-A [85% (95% CI, 63–87%) versus 70% (95% CI, 41–73%); *p* = 0.022], significantly higher median PP of VEGF-D [85% (95% CI, 60–90%) versus 60% (95% CI, 35–61%); *p* = 0.010], and significantly higher median IRS of VEGF-D [7 (95% CI, 5–9) versus 4 (95% CI, 3–6); *p* = 0.013]. Additionally, VEGFR1 staining intensity was significantly stronger in metastatic epithelial tumor cells (moderate-to-strong intensity in 88% of samples) than in the primary tumors (moderate-to-strong intensity in 74% of samples) (*p* = 0.046). In contrast to the VEGF-A and VEGF-D expressions, epithelial expression of VEGF-C was higher in the primary tumors than in the metastases (*p* = 0.010). Differences in other factors did not reach statistical significance (Table 2, Figs. 1, 3).

### qRT-PCR levels and correlations with immunohistochemical expression

In primary high grade tumors, we found a significant correlation between VEGF-D IRS and VEGF-D mRNA (correlation coefficient, r = 0.991, *p* = 0.004), VEGFR1 PP and VEGFR1 mRNA (r = 0.967, *p* = 0.014) and VEGFR3 SI and VEGFR3 mRNA (r = − 0.655, *p* = 0.04).

In omental metastases, the immunohistochemical expressions of all receptors correlated to the corresponding mRNA levels. Specifically, VEGFR1 mRNA level was significantly correlated with VEGFR1 IRS (r = 0.717; *p* = 0.009) and VEGFR1 PP (r = 0.780; *p* = 0.003); VEGFR2 mRNA was significantly correlated with VEGFR2 IRS (r = 0.679; *p* = 0.015) and VEGFR2 PP (r = 0.810; *p* = 0.001); and VEGFR3 mRNA was significantly correlated with VEGFR3 IRS (r = 0.667; *p* = 0.018) and VEGFR3 PP with (r = 0.661; *p* = 0.019). The VEGFR2 mRNA level was correlated with the percentage of VEGFR2 strong epithelial staining (r = 0.726; *p* = 0.011). The VEGFR3 mRNA level was correlated with the percentage of VEGFR3 strong epithelial staining (r = 0.854; *p* = 0.007), and with VEGFR3 stromal intensity (r = 0.599, Pearson’s test *p* = 0.04).

The mRNA levels of VEGF-A, VEGF-C, VEGF-D, VEGFR1, −R2 and **–**R3 did not reach statistical significance when primary tumors were compared to the related metastases.

### Relation of clinicopathological data to the expression of VEGFs and VEGF receptors

Ovarian cancer recurrence was associated with low expression of VEGF-A (*p* = 0.005) and VEGFR1 (*p* = 0.01). The high histological grade was related to low expression of VEGF-C (*p* = 0.006), VEGF-D (*p* = 0.04), VEGFR1 (*p* < 0.001), VEGFR2 (*p* = 0.008) and VEGFR3 (*p* = 0.004) in epithelial cells of the primary ovarian cancer.

When high grade serous and endometrioid tumors were analyzed as one group, low expression of VEGF-A (*p* = 0.010) and high expression of VEGFR3 (*p* = 0.037) were associated with the recurrence of cancer. Low VEGF-C (*p* = 0.026) was related to no residual tumor at primary surgery in high grade tumors.

In the whole study population, VEGF expression was related to the histological subtype. Strong expressions of VEGF-C, VEGFR1, VEGFR2, and VEGFR3 were most commonly found in tumors with mucinous histology, while the weakest expressions were observed in clear cell and endometrioid tumors (*p* = 0.033, *p* < 0.001, *p* = 0.027, *p* = 0.005, respectively). Of the studied parameters, serous tumors exhibited the largest variation.

Other clinical parameters were not significantly associated with the immunohistochemical data. We obtained parallel results when analyzing associations with Pearson’s chi-squared test and as continuous parameters with the Kruskal-Wallis test (Table 3).

**Table 3:** Relationship between immunohistochemical results and clinicopathological data

	Tumor histology	Grade	Stage	Recurrence
VEGF-A				
IRS	ns	ns	ns	0.005
VEGF-C				
IRS	0.033	0.006	ns	ns
PP	0.019	0.003	ns	ns
VEGF-D				
IRS	ns	0.040	ns	ns
PP	0.008	0.016	ns	ns
VEGFR1				
IRS	< 0.001	< 0.001	ns	0.010
PP	< 0.001	< 0.001	ns	0.003
SI	ns	0.008*	ns	ns
VEGFR2				
IRS	0.027	ns	ns	ns
PP	0.002	0.008	ns	ns
Stroma	ns	ns	ns	ns
VEGFR3				
IRS	ns	0.004	ns	ns
PP	0.005	0.004	ns	ns
SI	ns	0.026*	ns	ns

*p* values determined by Kruskal-Wallis test, *Pearson’s chi-squared test

*IRS* immunoreactive score, *PP* percentage of positively stained cells, *Recurrence* recurrence of cancer

### Overall survival and progression-free survival of the ovarian cancer patients

**Fig. 4:**
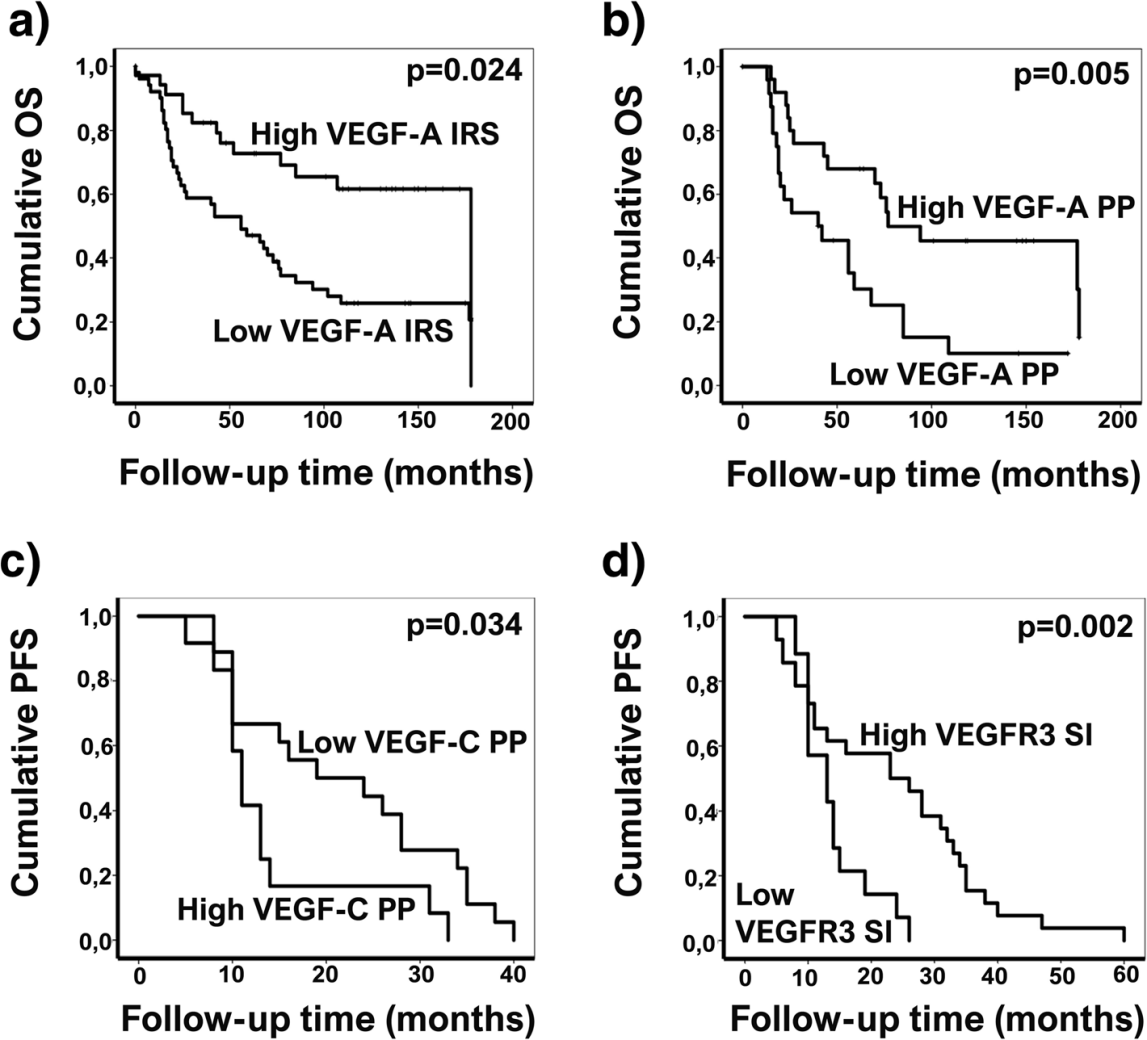
Univariate analysis of immunohistochemical biomarkers as prognostic factors in ovarian cancer patients. Poor overall survival (OS) was significantly associated with low VEGF-A immunoreactive score (IRS) (**a**) and with low VEGF-A percentage of positively stained cells (PP) in serous carcinoma (**b**). Short progression-free survival (PFS) was significantly associated with high VEGF-C PP (**c**) and low VEGFR3 staining intensity (SI) (**d**)

The median follow-up time was 65 months (range, 0–198 months). At the end of follow-up, 51 patients (59%) had died. The median OS was 77 months (95% CI, 51–102 months) and the 5-year survival rate was 57% (95% CI, 46–68%). In univariate analysis, low VEGF-A expression in the primary tumors predicted poor OS (*p* = 0.024). Among serous primary tumors, low VEGF-A PP was associated with short OS (*p* = 0.005) as well as in high grade serous and endometrioid tumors (*p* = 0.042) (Fig. 4). Univariate survival analysis revealed that poor OS was significantly predicted by advanced stage, presence of residual primary tumor, incomplete primary response to chemotherapy, and presence of ascites. In multivariate analysis, OS remained significantly associated with the presence of residual primary tumor (*p* < 0.001) and incomplete response to chemotherapy (*p* < 0.0001) (Table 4).

**Table 4:** Overall survival and progression-free survival according to immunohistochemical staining of VEGFs and receptors and clinical characteristics

Variable	Univariate analysis	Multivariate analysis		
		Hazard ratio,	95%Cl	*p*
Overall survival				
VEGF-A IRS	0.024			
VEGF-A PP (serous tumors)	0.005			
VEGF-A SI stroma	0.003			
Stage	< 0.001			
Ascites	0.046			
Primary residual tumor				
None				
< 1 cm	< 0.001	4.208	1.832–9.664	0.001
> 1 cm	< 0.001	4.392	2.159–8.938	< 0.001
Chemotherapy response				
Complete response				
Partial response	< 0.001	3.75	1.318–10.665	0.013
Stable disease	< 0.001	23.768	4.33–130.45	< 0.001
Progressive disease	< 0.001	12.896	4.67–35.612	< 0.001
Progression-free survival				
VEGF-C PP (serous tumors)	0.034			
VEGF-C SI stroma	0.036	0.131	0.021–0.815	0.029
VEGFR3 SI	0.002			
Stage	< 0.001	0.02	0.001–0.32	0.022
Ascites	0.028	2.152	1.018–4.55	0.045

*IRS* immunoreactive score, *PP* percentage of positively stained epithelial cells, *SI* staining intensity

PFS analysis included a total of 67 patients, of whom 42 (62%) experienced recurrence. The median PFS was 14 months (95% CI, 9–18 months). Short PFS was significantly associated with strong stromal staining of VEGF-C in primary tumors, in both univariate analysis (*p* = 0.036) and multivariate analysis (*p* = 0.029). In high grade serous and endometrioid tumors strong stromal VEGF-C was also related to short PFS (*p =* 0.021). Among high grade tumors, shorter PFS was associated with high VEGF-C (*p* = 0.034) and low VEGFR3 SI in epithelial cells of the primary tumor (*p* = 0.006). In both univariate and multivariate analysis, shorter PFS was significantly predicted by advanced stage (*p* = 0.022 in multivariate analysis) and the presence of ascites (*p* = 0.045 in multivariate analysis) (Table 4).

## Discussion

This is the first study to include the comprehensive analysis of a large panel of VEGFs and their receptors in the same patient population as well comparing primaries to the metastatic lesions, and link the results to the clinical outcome of the ovarian cancer patients. We found that expressions of VEGF-A, VEGF-D, and VEGFR1 were higher in omental metastases than in the related primary high grade serous tumors. Furthermore, poor OS was most strongly predicted by low VEGF-A expression in the primary high grade tumors, and short PFS was predicted by high VEGF-C expression.

Compared to primary tumors, the related ovarian cancer metastases showed increased expressions of the pro-angiogenic VEGF-A, VEGF-D, and VEGFR1 proteins. Metastatic tumors seemed to exhibit accumulation of these factors, with stronger expression in stromal tissue and vessel endothelium, enabling neovascularization and lymphangiogenesis. Moreover, VEGFR2, which binds VEGF-A, showed weaker expression in metastatic epithelial cells and stronger expression in stromal tissue and vascular endothelium. This is in line with previous findings that VEGFR2 and VEGFR3 are expressed on vascular endothelium, but to a lesser extent in malignant cells, in 13 common human solid cancer types, including ovarian cancer [24].

This result supports our previous finding in a smaller population that VEGF-A is present at higher levels in omental metastasis than in the primary tumors [25]. Gadducci et al. also demonstrated increased VEGF-A immunostaining in peritoneal metastatic lesions of ovarian cancer, but without showing prognostic significance or relation to clinical outcome [28].

Compared to the primary high grade serous tumors, related metastases showed lower expression of lymphangiogenic VEGF-C, and similar VEGFR3 expression. These results may suggest that angiogenesis is more active in distantly disseminated cancer metastasis, whereas lymphangiogenic VEGF-C predominantly acts more locally via paracrine mechanisms.

The findings of our present study, indicate that VEGF-A might be the most promising angiogenic factor for clinical use as a prognostic marker. Our data showed that low IRS and PP values for VEGFs and their receptors in high grade tumor epithelial cells were associated with ovarian carcinoma recurrence, and thus with more aggressive cancer. Low VEGF-A expression also predicted a short OS in the whole cohort, as well as in analyses considering only high grade serous carcinomas. These results might be explained by changes in the tumor microenvironment, hypoxia, and metabolic reprogramming. Hypoxia-inducible factor 1 (HIF1) is the main regulator of cellular responses to hypoxia, and induces angiogenesis. This regulation mechanism might not function equally in all tumors. Notably, strong cytoplasmic p53 expression has been associated with non-angiogenic malignant lung tumors [29, 30]. VEGF distribution could also vary between tumor tissues and circulation. Interestingly, we found that the lymphangiogenic factors VEGF-C and VEGFR3 were significantly correlated with PFS.

Earlier studies have yielded contradictory and heterogeneous results regarding angiogenic factor expression and prognosis in ovarian cancer. Nishida et al. concluded that high tumor expression of VEGF-C and VEGFR2 reflects ovarian carcinoma spread and poor prognosis [31], while Yokoyama et al. found that VEGF-D expression predicts poor OS in ovarian carcinoma [32]. In contrast, Engels et al. reported shorter PFS in cases of VEGF-negative ovarian tumors [26], and Shen et al. demonstrated a better OS among patients with low-VEGF-expressing ovarian tumors [33]. Additionally, Wimberg et al. found only low rates (18–43%) of positive VEGF receptor expression in primary ovarian cancer tissue [19]. Discrepancies between studies may be due to differences in analysis and utilized antibodies, or could be related to the specific carcinoma investigated, different tumor types, or patient groups with variable treatments.

The presently observed variation between histological subtypes of ovarian cancer may indicate that the VEGF pathway plays distinct biologic roles in these different groups. Findings in lung, breast, and gastric cancers suggest that the levels of VEGF autocrine loop activity have different impacts on distinct histologic cell phenotypes, potentially influencing the clinical outcome [18]. In our study, VEGF-C and VEGF receptors were most highly expressed in mucinous tumors. However, the number of mucinous carcinomas was low, as in the general population. Tumors with serous histology exhibited the largest variation; with well-differentiated papillary tumors seeming to show stronger expression of these factors. However, the statistics did not differ significantly between the total population and the high grade serous tumors.

In our present study, expression levels were semiquantitatively scored based on staining intensity and percentage of stained tumor cells, using the immunoreactive score as described [26]. This formula has been applied in various studies with slight modifications, since proposed by Stegner in 1986 [24, 34–37]. To elucidate the nuances behind the IRS, we also examined the separate factors PP and SI. The IRS has the advantage of being a single combining value, enabling comparison with other studies using the same formula. On the other hand, as a single value, it cannot accurately indicate all contributing factors, and the score formulation can vary among different studies [34, 37].

As a limitation of this study, the Western blot analysis was not performed. Antibodies used in this study were already tested with the Western blot by the vendors. We believe that with immunohistochemical stainings, we were able to detect the expression of growth factors and their receptors reliably, and analyse the exact location of these proteins in tumor tissue. In addition, qRT-PCR was performed to confirm the results in RNA level. However, in the future directions the Western blot analyses are needed.

There are not yet any tumor angiogenic biomarkers in clinical use, but antiangiogenic therapy development has raised an urgent need for prognostic and predictive biomarkers to improve patient selection, follow-up, and treatment effect monitoring. The first antiangiogenic agent to provide clinical benefit was the humanized monoclonal VEGF antibody bevacizumab. Since then, various VEGF neutralizing antibodies, multi-target tyrosine kinase inhibitors of the VEGF signaling pathway, and soluble VEGF receptors have entered clinical use, although none of them is yet in routine clinical use in ovarian cancer [4, 5, 7]. Our patient samples are from the era when bevacizumab was not in the clinical use.

VEGF inhibition can reduce the number of tumor vessels by impeding vascular sprouting and impairing endothelial survival, leading to vessel regression and thus normalizing the remaining vessel morphology. Our present results showed stronger VEGF-A, VEGF-D, and VEGFR1 expressions in related metastases, supporting the concept of using antiangiogenic treatments in disseminated carcinoma and for treating patients with incomplete primary surgical resection [4, 5, 32].

## Conclusions

Our results indicate that VEGF-A seems to be the most promising angiogenic marker in high grade serous ovarian cancer. VEGF-A expression was stronger in metastatic tumors, and low VEGF-A expression in primary tumors was associated with poor prognosis. In terms of lymphangiogenic factors, high VEGF-C expression and low VEGFR3 predicted shorter PFS. To improve antiangiogenic treatment efficacy, it would be beneficial to block both the angiogenic and lymphangiogenic pathways in ovarian cancer. Our present findings further suggest that it would be advantageous to examine a metastatic sample in addition to the primary tumor sample, to reveal the angiogenic status and improve the results of treatment with antiangiogenic therapies. Our data support the present and developing antiangiogenic treatment strategies, and suggest pathways for further investigation of angiogenic biomarkers.

## Data Availability

The datasets generated and analyzed in the current study are available from the corresponding author upon reasonable request.

## Abbreviations

Ang-2: Angiopoietin-2
bFGF: Basic fibroblast growth factor
CRC: Colorectal cancer
HIF1: Hypoxia-inducible factor
IRS: Immunoreactive score
mRNA: Messenger ribonucleic acid
ns: Non-significant
OS: Overall survival
PDGF: Platelet-derived growth factor
PFS: Progression-free survival
PP: Percentage of positively stained cells
qRT-PCR: Quantitative real-time polymerase chain reaction
SI: Staining intensity
TGF-β: Tumor growth factor β
